# Nutrient Excess Triggers the Expression of the *Penicillium chrysogenum* Antifungal Protein PAFB

**DOI:** 10.3390/microorganisms7120654

**Published:** 2019-12-04

**Authors:** Anna Huber, Hannah Lerchster, Florentine Marx

**Affiliations:** Institute of Molecular Biology, Biocenter, Medical University of Innsbruck, Innrain 80-82, 6020 Innsbruck, Austria

**Keywords:** antimicrobial proteins, *Penicillium chrysogenum*, *Penicillium chrysogenum* antifungal protein B (PAFB), nutrient, promoter, terminator, gene regulation, expression system

## Abstract

Nutrient limitation and nonfavorable growth conditions have been suggested to be major triggers for the expression of small, cysteine-rich antimicrobial proteins (AMPs) of fungal origin, e.g., the *Penicillium chrysogenum* antifungal protein (PAF), the *Aspergillus giganteus* antifungal protein (AFP), the *Aspergillus niger* antifungal protein (AnAFP). Therefore, these AMPs have been considered to be fungal secondary metabolite products. In contrast, the present study revealed that the expression of the PAF-related AMP *P. chrysogenum* antifungal protein B (PAFB) is strongly induced under nutrient excess during the logarithmic growth phase, whereas PAFB remained under the detection level in the supernatant of cultures grown under nutrient limitation. The efficiency of the *pafB*-promoter to induce PAFB expression was compared with that of two *P. chrysogenum* promoters that are well established for recombinant protein production: the *paf*-promoter and the xylose-inducible promoter of the xylanase gene, *xylP*. The inducibility of the *pafB*-promoter was superior to that of the *xylP*-promoter yielding comparable PAFB amounts as under the regulation of the *paf*-promoter. We conclude that (i) differences in the expression regulation of AMPs suggest distinct functional roles in the producer beyond their antifungal activity; and (ii) the *pafB*-promoter is a promising tool for recombinant protein production in *P. chrysogenum*, as it guarantees strong gene expression with the advantage of inducibility.

## 1. Introduction

Fungi with sequenced genomes that belong to the class Eurotiomycetes contain at least one gene coding for antimicrobial proteins (AMPs) [1]. These proteins are small in size (~6.5 kDa), cysteine-rich and amphipathic, and are secreted into the culture broth by their producer strains. Their compact disulfide-stabilized tertiary structure—containing five β-strands—renders them highly stable against adverse environmental conditions [2,3,4,5].

AMPs efficiently inhibit the growth of human and plant pathogenic fungal species when applied at low micromolar concentrations and those acting in a fungicidal way, lower the risk of resistance development. Extensively studied examples are AMPs from *Penicillium chrysogenum* (*P. chrysogenum* antifungal protein (PAF) and *P. chrysogenum* antifungal protein B (PAFB)) [6,7], *Neosartorya fischeri* (*N. fischeri* antifungal protein (NFAP) and *N. fischeri* antifungal protein 2 (NFAP2)) [8,9], *Penicillium digitatum* (*P. digidatum* antifungal protein B (AfpB)) [10], *Penicillium expansum* (*P. expansum* antifungal proteins A, B, C (PeAfpA, PeAfpB and PeAfpC)) [11], *Aspergillus giganteus* (*A. giganteus* antifungal protein (AFP)) [12,13] and *Aspergillus niger* (*A. niger* antifungal protein (AnAFP)) [14]. Most of them show no cytotoxicity to mammalian cells in vitro [10,11,15,16,17,18] and in vivo [16,19].

AMPs are therefore considered as promising candidates for the development of novel antifungal treatment strategies in medicine and agriculture [9].

Many studies have been published and provide insight into the AMPs specificity, their structure and their antimicrobial mode of action. However, limited information is available on the expression regulation of the respective encoding genes and the cultivation conditions that trigger production of these biomolecules by their producers. AMP yields vary significantly between different fungal species and cultivation conditions. PAF, for example, is secreted in high amounts by *P. chrysogenum* [15], whereas the quantities of NFAP appear comparably low in the culture supernatant of *N. fischeri* [8]. The knowledge how AMP gene expression is regulated is important for two reasons: (i) to understand the AMPs’ function for the host; and (ii) to identify culture conditions to improve AMP production and reach protein yields that are sufficient for their experimental use to investigate their structure and mode of action and for their applicability in vitro and in vivo. For high-yield AMP production, our group has established a *P. chrysogenum*-based expression system that works under the control of the promoter of the PAF encoding gene (*paf*) [20]. This gene is strongly induced in minimal medium under nutrient-limiting conditions when the culture has entered the nonexponential growth phase. Nutrient limitation and nonfavorable growth conditions e.g., heat shock or pH-stress were reported to trigger the production of other AMPs, like the *A. gigenteus* AFP or the *A. niger* AnAFP [21,22]. This expression regulation led to the hypothesis that ascomycetes AMPs cover—in addition to their growth inhibition activity—additional regulatory functions in the producing fungi; for example, they play a role in development [21,23], apoptosis [24], autolysis and autophagy [25].

In contrast to this assumption, the expression of the PAF-related *P. chrysogenum* antifungal protein PAFB was found to be not induced under these cultivation conditions [7]. The *pafB*-mRNA was only slightly expressed in minimal medium and the protein PAFB remained under the detection level in the culture supernatant. This necessitated the application of the *P. chrysogenum*-based expression system for high-yield and high-quality production of PAFB for structural and functional analyses [7]. PAFB revealed a PAF-like compact β-folded structure, containing five β-strands connected by four flexible loops and an “*abcabc*” disulphide bond pattern. Functional studies identified human pathogenic fungal species such as *Aspergillus fumigatus, Candida albicans* or *Trichophyton rubrum* and model organisms, such as *Neurospora crassa*, to be highly susceptible against low doses (μM) of PAFB and PAF [7].

In the present study, we searched for cultivation conditions triggering PAFB expression and secretion into the culture broth of *P. chrysogenum wild-type* (*wt*). Interestingly and unexpectedly, we found an increased and sustained induction of the *pafB* gene during the exponential growth phase under high nutrient availability. The differences in the regulation of PAF and PAFB expression let us hypothesize that these two AMPs cover diverse functions in *P. chrysogenum* beyond their antifungal activity. Applying nutrient abundance, we could purify high amounts of the native PAFB from the culture supernatant. The efficiency of the *pafB*-promoter to induce PAFB expression was further compared with that of two already well established promoter systems derived from *P. chrysogenum*: the *paf*-promoter and the xylose-inducible promoter of the xylanase gene *xylP* [26], revealing a clear superiority of the *pafB*-promoter. The inducibility of the *pafB*-promoter by nutrient excess and the high transcription efficiency suggests this promoter to be a suitable tool for high-yield production of recombinant proteins in *P. chrysogenum*.

## 2. Materials and Methods

### 2.1. Strains, Media and Cultivation Conditions

Media and strains used in this study are listed in Supplementary Tables S1 and S2. Conidia of all *P. chrysogenum* strains were generated on solid *P. chrysogenum* minimal medium (PcMM) at 25 °C for 72–96 h and the spores were harvested in spore buffer (0.9% NaCl (*w*/*v*), 0.01% Tween 80 (*v*/*v*)). To monitor RNA and protein expression and for protein generation 2 × 10^8^ conidia of the respective *P. chrysogenum* strains were cultivated in 200 mL medium at 25 °C for up to 96 h. PAFB expression was monitored in *P. chrysogenum wt* grown in PcMM containing up to fourfold molar excess of all compounds (1×, 2×, 3× and 4× PcMM, respectively) and in complete medium (CM) containing onefold and fourfold molar excess of all compounds (1× CM and 4× CM). To determine the impact of the different compounds of PcMM on *pafB* expression, *P. chrysogenum wt* was grown in PcMM, in which the single compounds were supplemented in fourfold excess, respectively: KCl (26.8 mM), FeSO_4_ × 7H_2_O (0.72 mM), MgSO_4_ × 7H_2_O (8 mM), NaNO_3_ (141.2 mM) or sucrose (232 mM).

For the determination of the promoter efficiency the *P. chrysogenum* mutant *pafB^paf_promoter^* was cultivated in 1× PcMM containing 2% sucrose, the mutant *pafB^xylP_promoter^* in 1× PcMM containing 2% xylose and the *P. chrysogenum wt* in 4× PcMM containing 8% sucrose as sole carbon source, respectively. *Bacillus subtilis* was grown on lysogeny broth (LB) agar plates.

### 2.2. Cloning Vector Construction and Fungal Transformation for Promoter/Terminator Exchange

Oligonucleotides used for cloning are indicated in Supplementary Table S3. The bipartite marker technique was used for generating the *pafB^paf_promoter^, pafB^paf_terminator^* and the *pafB^xylP_promoter^* mutants, respectively. For each strain, the *P. chrysogenum wt* was cotransformed with two polymerase chain reaction (PCR) fragments (Figure S1).

For the generation of the *pafB^paf_promoter^* strain, *P. chrysogenum wt* was cotransformed with the PCR fragments A and B. To generate fragment A, gDNA of *P. chrysogenum wt* was amplified using the primer pair pafB3′*Not*I_rev and pafB_*Bgl*II_fw. The PCR product was digested using the enzymes *Bgl*II and *Not*I and ligated into the plasmid pSK275*pafB* [7]. From this plasmid, a fragment containing the *paf* 5′untranslated region (UTR), the *pafB* gene and the *pafB* 3′UTR was released by using the enzymes *Not*I and *Xba*I and ligated into the plasmid pD-NAT1 [27], resulting in plasmid pD-NAT1*_pafB^paf_promoter^*. Fragment A (4767 bp) was amplified by the primer pair Onat2 and 3′pafb flanken nested_fw.

To generate fragment B, gDNA of *P. chrysogenum wt* was amplified using the primer pair pafB5_*BamH*I_rev and pafB5_*Pst*I_fw. The fragment was digested and ligated into plasmid pD-NAT1. Fragment B (2883 bp) was amplified from this plasmid by using the primer pair Onat1 and 5′pafb fl nested_fw.

For the generation of the *pafB^xylP_promoter^* strain, *P. chrysogenum wt* was cotransformed with the PCR fragments B & C. Fragment C was generated by amplifying the *xylP*-promoter sequence from plasmid px-ergA [28] using the primer pair Xylp_*Xba*I_rev_B and Xylp_*Bgl*II_fw_B. The PCR fragment was digested using the enzymes *Xba*I and *Bgl*II and the paf-5′UTR region in plasmid pD-NAT1*_pafB^paf_promoter^* was exchanged by the *xylP*-promoter sequence ending up in plasmid pD-NAT1*_paf^xylP_promoter^*. Fragment C (4729 bp) was amplified by the primer pair Onat2 and 3′pafb flanken nested_rev and fragment B was generated as described above.

The *pafB^paf_terminator^* mutant was generated by cotransforming the *P. chrsogenum wt* with the fragments D and E. Fragment D was generated by amplifying gDNA of the *P. chrysogenum wt* strain with the primer pair pafB5_*Pst*I_fw and pafB_*Sma*I_rev. The purified PCR fragment was digested by *Pst*I and *Sma*I and ligated into plasmid pSK275*pafB* [7], resulting in plasmid pSK275*pafB*^*paf3*′*pafb5*′^. The plasmid pSK275*pafB*^*paf3*′*pafb5*′^ was used in PCR reaction together with the primer pair, pafB5_*Pst*I_fw and 3′paf_*BamH*I_rev. This fragment was digested using the enzymes *Pst*I and *BamH*I and ligated into plasmid pD-NAT1, resulting in plasmid pD-NAT1*_pafB^paf_terminator^*. pD-NAT1*_pafB^paf_terminator^* was used as template to generate the transformation fragment D (3677 bp) by using the primers Onat1 and 5′pafb fl nested_fw.

Fragment E was generated by amplifying gDNA of *P. chrysogenum wt* by the primer pair pafB3_*Xba*I_fw and pafB3′*Not*I_rev. After digesting the fragment with the enzymes *Not*I and *Xba*I, it was ligated into plasmid pD-NAT1*_pafB^paf_terminator^,* resulting in plasmid pD-NAT1*_pafB^paf_terminator^*^/pafB5′UTR^. Plasmid pD-NAT1*_pafB^paf_terminator^*^/pafB5′UTR^ was used together with the primer pair, Onat2 and 3′pafb flanken nested_rev, in a PCR reaction to generate fragment E (2348 bp).

PCR fragments A and B, B and C, and D and E shared a 400-bp overlap within the *nat1* cassette, which served as a potential recombination site during transformation. The transformation of *P. chrysogenum* protoplast with 5 µg DNA per fragment was carried out according to Cantoral et al. (1987) [29]. Transformants were single-spored three times on PcMM agar supplemented with 200 μg/mL nourseothricin (Jena Bioscience, Jena, Germany).

Positive transformants were analyzed by Southern blotting for homologous integration (Figure S1).

### 2.3. Southern Blot Analysis

Southern blot analysis was performed to verify locus specific and single copy integration in the mutants *pafB^paf_promoter^*, *pafB^xylP_promoter^* and *pafB^paf_terminator^*. Genomic DNA was extracted according to Zadra et al. (2000) [26].

Per lane, 2 μg gDNA of the respective mutant and the *wt*—digested using the respective restriction enzymes—was size-fractionated on a 0.8% (*w*/*v*) agarose gel. The DNA was transferred onto Hybond-N membranes (Amersham Biosciences, Little Chalfont, UK) and hybridized with digoxigenin (DIG)-labelled probes (Roche Diagnostics, Mannheim, Germany), specific for the nourseothricin-acetyltransferase gene (*nat1*) and the *pafB* gene, respectively. The probes were generated from pSK275*pafB* or pD-NAT1 by PCR using the oligonucleotides opafb_fw/opafb_rev2 or Onat1/Onat2.

### 2.4. Northern Blot Analysis

Northern blot analysis was used to monitor the mRNA expression of *pafB* and *paf* as well as the expression of genes encoding the ribosomal proteins S5 and S6. Total RNA was extracted from mycelia of the respective *P. chrysogenum* strains using TRI Reagent^®^ (Sigma-Aldrich, Vienna, Austria). Ten µg RNA per lane were loaded on 1.2% formaldehyde-agarose gels, blotted onto Hybond-N membranes (Amersham Biosciences) and hybridized with DIG-labelled probes (Roche). For generation of *pafB* and *paf* specific hybridization probes, the primer pairs opafB_without prepro_fw/opafB_rev2 and opaf_without prepro_fw/opaf_rev were used in the PCR reaction, respectively. For probes to specifically detect gene expression of the ribosomal protein S5, the primer pair 40S rib_protein_S5_rev and 40S rib_protein_S5_fw, and for protein S6, the primer pair 40S rib_protein_S6_rev and 40S rib_protein_S6_fw were used.

### 2.5. Protein Expression, Purification and Verification

Recombinant PAF and PAFB were generated by cultivating the strains *P. chrysogenum paf* and *P. chrysogenum pafB* in 1× PcMM, respectively. Purification was performed as recently described [20]. Native PAFB was expressed in 4× PcMM by the *P. chrysogenum wt* strain and purified as described [7,20]. The higher positive net charge (+5.2 at pH 7) of PAFB enabled the separation of PAFB from PAF by elution at a higher salt concentration (500 mM NaCl).

PAFB expression by the mutant *pafB^paf_promoter^* was performed in 1× PcMM. The PAFB purification strategies are depicted in Supplementary Methods. Electro-spray ionization mass spectrometry (ESI-MS) was used to determine protein mass and to verify the identity of purified PAFB (Protein Micro-Analysis Facility at the Medical University of Innsbruck).

### 2.6. SDS-PAGE and Western Blotting

For the detection of PAFB and PAF in the culture broth, 25 µL of cell-free culture supernatant were separated on a 18% (*w*/*v*) tris-glycine sodium dodecyl sulfate (SDS)-polyacrylamide gel and then transferred to nitrocellulose membranes (Bio Trace, Pall, Port Washington, NY, USA). After blotting, membranes were blocked for 2 h in PBS/0.3% Tween (*v*/*v*)/3.0% skim milk powder (*w*/*v*) and then incubated overnight at 4 °C with anti-PAFB (α-PAFB) or anti-PAF (α-PAF) antiserum (1:1000 in PBS/0.3% Tween (*v*/*v*)/1.0% skim milk powder (*w*/*v*)). After washing in PBS/0.3% Tween (3 × 10 min), the membranes were incubated with alkaline phosphatase conjugated goat anti-rabbit secondary antibody (Sigma), diluted 1:10,000 in PBS/0.3% Tween (*v*/*v*)/1.0% skimmed milk powder (*w*/*v*) for 1 h at room temperature. The membranes were washed and protein immunocomplexes were visualized using nitro blue tetrazolium chloride (NBT)/5-bromo-4-chloro-3-indolyl-phosphate (BCIP) (Promega, Madison, WI, USA).

### 2.7. Agar Diffusion Test

To monitor the penicillin content in the fungal culture, broth agar diffusion tests were performed. Four colonies of *B. subtilis* were harvested from overnight agar plates and transferred into 2 mL of LB medium. One-hundred µL of the bacterial suspension were added to 10 mL LB medium and poured on a petri dish (diameter = 13.5 cm) containing 100 mL LB-agar. The plate was incubated for 1 h at 37 °C, then the liquid was removed and holes were punched into the agar.

Cell-free supernatants of 24–120-h-old *P. chrysogenum wt* cultures grown in 1× PcMM or 4× PcMM were added to the holes. The amount of the loaded culture broth was adjusted to the mycelial dry weight and calculated according to the following formula, 250/gDW × 6. To prove the presence of penicillin, 250 µL of cell-free culture supernatant (1× PcMM, 120 h cultivation) were treated for 1 h at 30 °C with 1000 U penicillinase (TCI). As a control, 2.5 µg and 1.2 µg penicillin G sodium salt (Sigma) were used instead of the culture broth and treated with 1000 U penicillinase in a total volume of 250 µL. The samples were then used in agar diffusion assays. As controls, untreated penicillin (1.2 and 2.5 µg), untreated supernatant (250 µL, 1× PcMM, 120 h cultivation) and 1000 U penicillinase were used, respectively. Plates were incubated overnight at 37 °C and inhibition zones were evaluation.

### 2.8. Microscopy and Image Processing

The validation of pellet morphology was done with an inverted Leica DM IL LED microscope (Leica Microsystems, Wetzlar, Germany) and imaging was performed with an AxioCam MR3 camera (Carl Zeiss GmbH, Oberkochen, Germany). Image processing and editing was achieved with the programs Axio Vision software (Carl Zeiss GmbH), GNU Image Manipulation Program (GIMP, version 2.8.201) and Microsoft PowerPoint (Microsoft Corp., Redmond, WA, USA).

### 2.9. Statistical Analysis

Statistical analysis was performed using Microsoft Excel 2010 software (Microsoft Corp.). A two-sample *t*-test with equal variance and one-tailed distribution was applied to determine the *p*-values.

## 3. Results

### 3.1. The Expression of pafB Is Induced by Nutrient Excess

**Figure 1:**
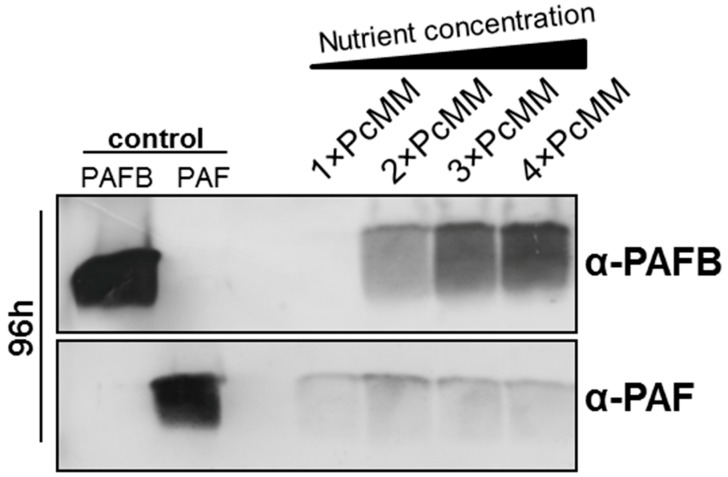
Expression of the *P. chrysogenum* antifungal protein (PAF) and *P. chrysogenum* antifungal protein B (PAFB) in minimal medium (PcMM) containing increasing nutrient concentrations. *P. chrysogenum wt* was cultivated in 1×, 2×, 3× or 4× PcMM, respectively, at 25 °C for 96 h. Western blot: Culture supernatants (25 μL per lane) were loaded on a 18% (*w*/*v*) sodium dodecyl sulfate (SDS) polyacrylamide gel and size fractionated proteins were transferred on a nitrocellulose membrane. Recombinant PAFB and PAF (1 μg per lane) served as controls, respectively. A polyclonal antibody (α-PAFB or α-PAF) was used for specific PAFB and PAF detection in a 1000-fold dilution, respectively.

We have recently reported that the protein PAFB remained under the limit of detection in the culture supernatant, although the *pafB* gene expression was induced after 48 h of cultivation in standard PcMM (1× PcMM) [7]. To identify conditions that trigger PAFB expression, we tested different media with variable nutrient compositions. In minimal media containing a two-to-four-fold nutrient excess (2×, 3× and 4× PcMM), PAFB expression was strongly induced and the protein amount in the supernatant correlated with the increasing nutrient concentration (Figure 1). In contrast, *paf* gene expression peaked after 72 h of cultivation in 1× PcMM [7] and PAF-production could not be further increased by high nutrient availability (Figure 1). It should be noted that the salts added with the trace element solution or the KPO_4_-buffer remained single-concentrated in 2×–4× PcMM, respectively. Therefore, these components could be excluded to trigger *pafB* transcription.

Based on these findings, we conclude that PAFB expression differs from that of other AMPs like PAF (*P. chrysogenum*), AFP (*A. giganteus*) and AnAFP (*A. niger*), which are strongly induced under nutrient starvation conditions [25].

**Figure 2:**
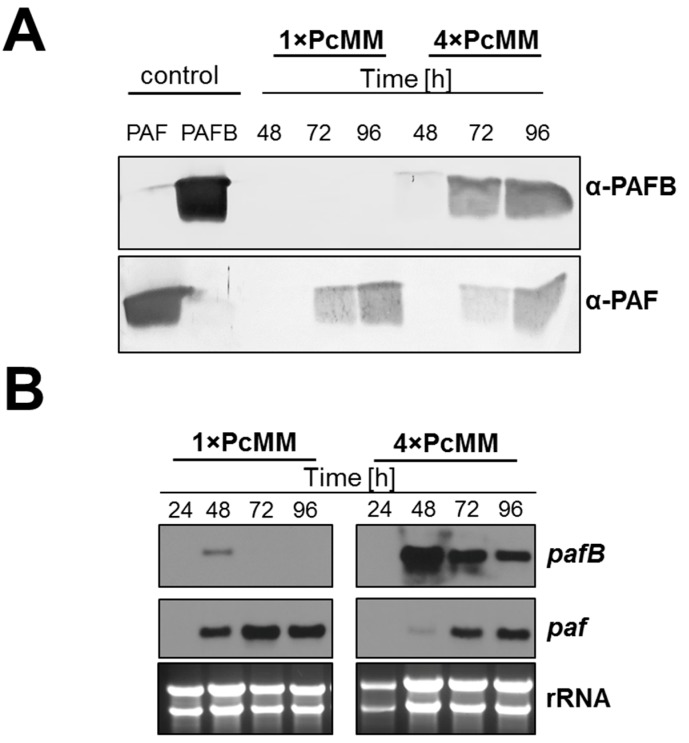
Comparison of PAFB and PAF expression on mRNA and protein level in *P. chrysognum wt* under low nutrient (1× PcMM) and nutrient excess conditions (4× PcMM). (**A**) Western blot: Samples were taken after 48, 72 and 96 h of cultivation at 25 °C. Culture supernatants (25 μL per lane) were loaded on a 18% (*w*/*v*) SDS polyacrylamide gel and size fractionated proteins were transferred on a nitrocellulose membrane. A polyclonal antibody (α-PAFB or α-PAF) was used for specific PAFB and PAF detection in a 1000-fold dilution. As control, 1 µg purified PAFB or PAF were loaded. (**B**) Northern blot: Samples to monitor mRNA expression were taken after 24, 48, 72 and 96 h of incubation at 25 °C. Ten μg total RNA were loaded per lane on a 1.2% denaturing agarose gel, blotted and hybridized with a *pafB* or *paf*-specific digoxigenin (DIG)-labelled probe. Ethidium bromide-stained rRNA (26S and 18S) is shown as loading control.

To evaluate the expression pattern of PAFB on gene transcript and protein level, we performed time-course experiments with *P. chrysogenum wt* grown in 1× PcMM and 4× PcMM. In 4× PcMM, PAFB could be detected after 48 h of inoculation and the protein amount steadily increased with the cultivation time (up to 96 h) (Figure 2A). Gene transcription preceded protein production, peaked after 48 h of cultivation and remained elevated during the applied time course (up to 96 h). In contrast the *pafB*-mRNA signal from 1× PcMM culture conditions was much weaker at the cultivation time of 48 h and disappeared 24 h later (Figure 2B). For comparison, the PAF content in the supernatant differed not significantly between high (4× PcMM) and low (1× PcMM) nutrient conditions (Figure 2A). The *paf*-mRNA expression reached a maximum after 72 h of cultivation in 1× PcMM, whereas in 4× PcMM, gene expression was delayed for 24 h (Figure 2B).

Next, we wanted to identify which components of PcMM induce *pafB* expression in *P. chrysogenum wt*. To answer this question, we supplemented 1× PcMM with each of the following compounds, KCl, MgSO_4_, FeSO_4_, sucrose or NaNO_3_ in 4-fold concentration, respectively.

**Figure 3:**
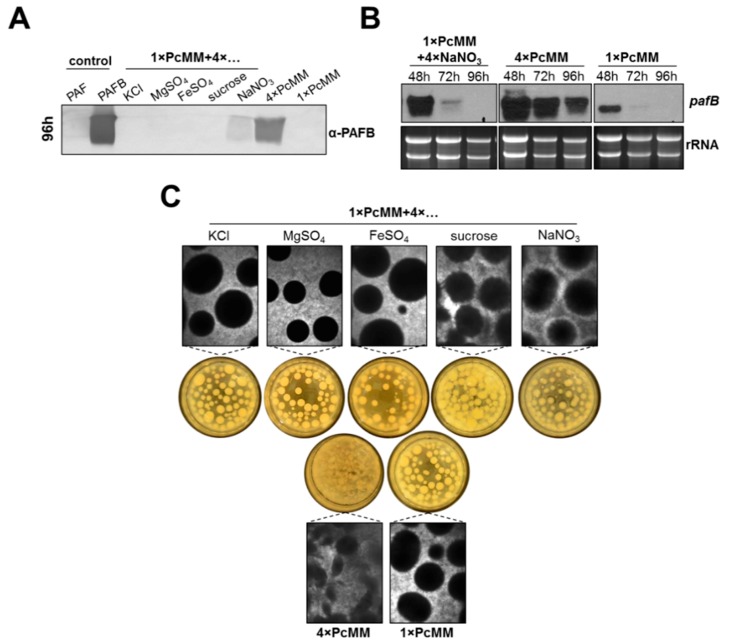
Impact of PcMM compounds on *pafB* expression and fungal culture morphology. *P. chrysogenum wt* was cultivated in 1× PcMM, 4× PcMM and 1× PcMM supplemented with 4× KCl, 4× MgSO4, 4× FeSO4, 4× sucrose or 4× NaNO_3_, respectively. (**A**) Western blot: PAFB secretion into culture supernatant was monitored after 96 h of cultivation. Culture supernatants (25 μL per lane) were loaded on a 18% (*w*/*v*) SDS polyacrylamide gel and size fractionated proteins were transferred on a nitrocellulose membrane. A polyclonal antibody (α-PAFB) was used for specific PAFB detection in a 1000-fold dilution. As control, 1 µg purified PAFB and PAF were loaded. (**B**) Northern blot: mRNA expression in *P. chrysogenum wt* grown in 1× PcMM + 4× NaNO_3_ was compared to that cultivated in 1× PcMM and 4× PcMM. Expression was analyzed after 48, 72 and 96 h. Ten μg total RNA per lane were loaded on a 1.2% denaturing agarose gel, blotted and hybridized with a *pafB*-specific DIG-labelled probe. Ethidium bromide-stained rRNA (26S and 18S) is shown as loading control. (**C**) Morphology of fungal pellets after 96 h of cultivation under low and high nutrient conditions.

Western blot analysis revealed that increased nitrogen availability triggered PAFB production (96 h cultivation), although the protein amount in the supernatant remained significantly lower compared to that in 4× PcMM. The excess of the other compounds did not induce protein production (Figure 3A). We therefore monitored *pafB*-mRNA expression under increased nitrogen availability in a time course of 48–96 h of cultivation. Transcription induction after 48 h of cultivation could be observed with fourfold excess of NaNO_3_; however, the amount of *pafB*-mRNA rapidly decreased after this time point and resulted in only a mild accumulation of PAFB in the supernatant after 96 h. In contrast, high and sustained amounts of *pafB* transcripts were detectable in 4× PcMM, which was reflected in a significantly increased PAFB amount in the culture broth (Figure 3B). We therefore conclude, that not only the upregulation of transcription but also a sustained transcription over time are necessary to reach high PAFB yields in the culture broth.

Interestingly, the morphology of the submerse culture grown in 4× PcMM markedly differed from that grown under 1× PcMM and 1× PcMM supplemented with 4-fold excess of the single compounds, respectively. In 4× PcMM the pellets appeared less dense, in comparison to the other two growth conditions where more defined and compact pellets were formed (Figure 3C).

**Figure 4:**
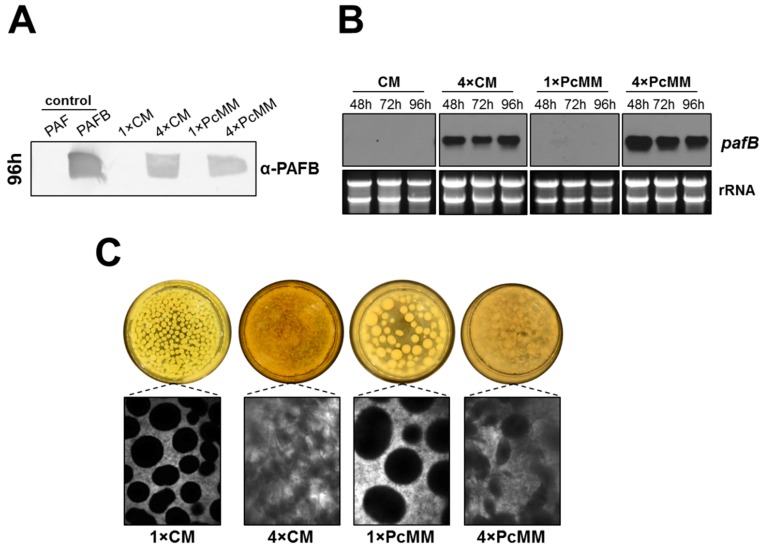
Impact of complete medium on *pafB* expression and fungal culture morphology. The *pafB* expression was compared between *P. chrysogenum wt* grown up to 96 h at 25 °C in 1× CM, 4× CM, 1× PcMM and 4× PcMM, respectively. (**A**) Western blot: PAFB secretion into the culture supernatant after 96 h of cultivation. Culture supernatants (25 μL per lane) were loaded on a 18% (*w*/*v*) SDS polyacrylamide gel and size fractionated proteins were transferred on a nitrocellulose membrane. A polyclonal antibody was used for specific PAFB detection in a 1000-fold dilution. As control, 1 µg purified PAFB and PAF were loaded. (**B**) Northern blot: *pafB*-mRNA expression. Samples were taken after 48, 72 und 96 h of cultivation. Ten μg total RNA per lane were loaded on a 1.2% denaturing agarose gel, blotted and hybridized with a *pafB*-specific DIG-labelled probe. Ethidium bromide-stained rRNA (26S and 18S) is shown as loading control. (**C**) Morphology of fungal pellets after 96 h of cultivation in the respective medium.

Our results let us assume that not only the excess of nutrients but also the combination of compounds in the medium is needed for the induction of prolonged *pafB* expression. We were therefore interested if *pafB* is expressed in a nutrient-rich complete medium (CM) like in 4× PcMM. In 1× CM, we could neither identify PAFB in the culture supernatant after 96 h of cultivation nor detect *pafB* gene transcripts. In contrast, in 4× CM, PAFB was secreted into the culture supernatant in similar amounts as detected in 4× PcMM (Figure 4A). This result correlated with the increased amount of *pafB*-mRNA over the investigated time (48–96 h) (Figure 4B). Interestingly, the fungal morphology in 4× CM was similar to that in 4× PcMM, where fluffy mycelium was formed, instead of dense pellets as observed in the single concentrated media, respectively (Figure 4C).

### 3.2. PAFB Is Expressed during Exponential Growth

To provide evidence for the fitness of the fungal culture under *pafB*-inducing vs. noninducing conditions, we evaluated parameters such as biomass, secondary metabolite production and expression of genes encoding ribosomal proteins.

**Figure 5:**
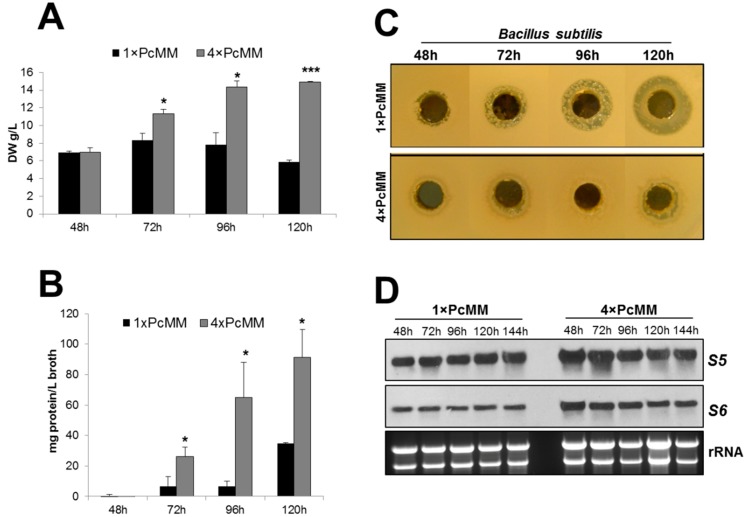
Parameters reflecting the fungal fitness under *pafB*-inducing and noninducing conditions. *P. chrysogenum wt* was grown in 1× PcMM and 4× PcMM at 25 °C, respectively. Samples were taken after 48, 72, 96 and 120 h to monitor biomass, extracellular protein and penicillin production. Expression of genes coding for ribosomal protein S5 and S6 was analyzed up to 144 h. (**A**) Fungal biomass: Biomass is indicated as gram dry weight per liter (DW g/L). Values are given as mean ± SD (*n* = 3). (**B**) Extracellular protein production: Secretion of extracellular proteins is indicated as mg protein per liter culture broth (mg protein/L broth). Values are given as mean ± SD (*n* = 3). (**C**) Penicillin production: Penicillin production was tested on *B. subtilis*. The amount of culture broth tested, was related to the dry weight of the biomass. Plates were evaluated after 24 h of incubation (**D**) Northern blot: mRNA expression of ribosomal proteins S5 and S6. Ten μg total RNA per lane were loaded on a 1.2% denaturing agarose gel, blotted and hybridized with S5 and S6 specific DIG-labelled probes, respectively. Ethidium bromide-stained rRNA (26S and 18S) is shown as loading control. *P*-values in (**A**,**B**) were determined to compare the biomass production and the extracellular protein level between 1× PcMM and 4× PcMM conditions, respectively. * *p* ≤ 0.05, *** *p* ≤ 0.0005.

We determined the fungal biomass and observed an increase of dry weight under *pafB*-inducing conditions (4× PcMM) from 48 to 120 h, reflecting the exponential growth phase during this cultivation period. Under *pafB* noninducing conditions (1× PcMM), in contrast, the biomass reached a maximum after 72 h of cultivation and then diminished with prolonged incubation time indicating the start of the stationary growth phase (Figure 5A). This paralleled well with significantly more extracellular proteins secreted into 4× PcMM supernatant compared to 1× PcMM culture broth over a time periode of 120 h. These results pointed at a highly active fungal metabolism under *pafB*-inducing culture conditions (Figure 5B).

To further substantiate our assumptions that PAFB is produced under favourable growth conditions, we investigated the production of secondary metabolites like penicillins, which are strongly induced under stress conditions when resources are low [30,31]. We therefore compared the penicillin production of *P. chrysogenum* grown in 1× PcMM and 4× PcMM. For this, we applied an agar diffusion test and exposed the penicillin-sensitive gram positive *B. subtilis* to conditioned cell-free supernatant collected at various time points from the respective *P. chrysogenum* cultures. The growth inhibition zones around the holes containing the conditioned 1× PcMM supernatant indicated the presence of a compound that inhibits the growth of *B. subtilis*. The strongest inhibition was observed with the sample collected from *pafB*-repressing condition after 120 h of cultivation. The inhibition zone diameter reached 20 mm. The supernatnat from cultures grown in 4× PcMM, in contrast, did not cause any distinct inhibition zones (Figure 5C). To verify the presence of penicillin in the culture broth, we treated the *P. chrysogenum* conditioned cell-free supernatant with β-lactamase—an enzyme that specifically degrades penicillins [32], before applying it in the agar diffusion assay. Indeed, the supernatant had lost antibacterial activity after β-lactamase treatment (Figure S2). The absence of antibiotic production under *pafB*-inducing conditions further substantiate that the fungal culture had not entered the stationary growth phase and has therefore not reached starvation conditions.

Finally, we tested the expression of genes encoding the ribosomal proteins S5 and S6 to evaluate the physiological state of the culture. It is well known that nutrient starvation may lead to the induction of autophagy, which is reflected by the downregulation of ribosomal genes [33]. When comparing the expression of genes coding for the ribosomal proteins S5 and S6 in 1× PcMM and 4× PcMM, we could not detect any substantial change in the transcriptional levels of both genes indicating the absence of autophagy under both cultivation conditions (Figure 5D).

Taken together, our results indicate that PAFB is produced in *P. chrysogenum wt* cultures with high metabolic activity. This stands in contrast to the reported culture conditions under which the *P. chrysogenum* PAF, the *A. giganteus* AFP and the *A. niger* AnAFP are produced, and for which a role in nutrition, apoptosis or autophagy has been suggested [24,25].

### 3.3. Increased and Sustained pafB Gene Expression Is Promoter-Regulated

**Figure 6:**
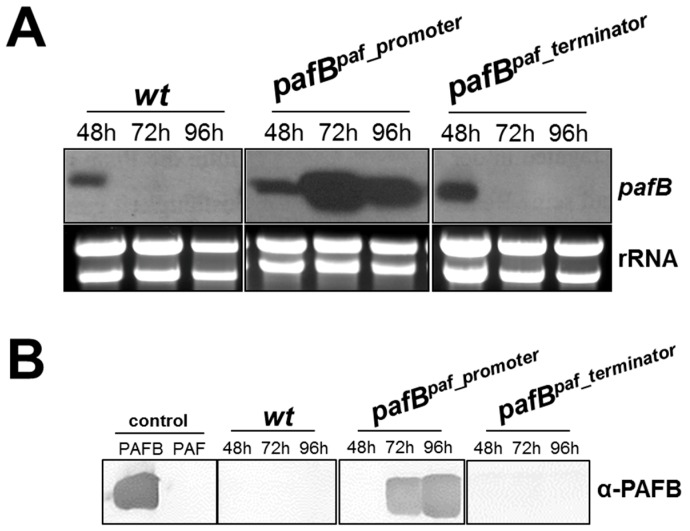
Expression of *pafB* in *P*. *chrysogenum* *wt* and in the mutants *pafB^paf_promoter^* and *pafB^paf_terminator^*. Strains were cultivated in 1× PcMM up to 96 h at 25 °C. Samples were analyzed after 48, 72 and 96 h. (**A**) Northern blot: *pafB*-mRNA expression in the mutants and the *wt*. Ten μg total RNA per lane were loaded on a 1.2% denaturing agarose gel, blotted and hybridized with a *pafB*-specific DIG-labelled probe. Ethidium bromide-stained rRNA (26S and 18S) is shown as loading control. (**B**) Western blot: Secretion of PAFB into the culture supernatant. Culture supernatants (25 μL per lane) were loaded on a 18% (*w*/*v*) SDS polyacrylamide gel and size fractionated proteins were transferred on a nitrocellulose membrane. A polyclonal antibody was used for specific PAFB detection in a 1000-fold dilution. As control, 1 µg purified PAFB and PAF were loaded.

The low amount of *pafB* transcripts in 1× PcMM could result from low transcription efficiency due to promoter-based gene repression or from mRNA instability regulated by the *pafB* 3′UTR region. We therefore exchanged the *pafB*-promoter by the *paf*-promoter and the *pafB* 3′UTR by the *paf* 3′UTR, respectively, generating the mutants *P. chrysogenum pafB^paf_promoter^* and *P. chrysogenum pafB^paf_terminator^*. Both mutants were verified to contain a *pafB*-locus specific single integration of the respective transforming DNA, replacing the *wt pafB* gene (Supplementary Figure S1a,c). The *pafB* expression in these mutants was compared with that of the *P. chrysogenum wt*. The exchange of the *pafB*-promoter by the *paf*-promoter resulted in a *pafB* transcription pattern resembling that of *paf*. The significantly induced *pafB*-mRNA transcription peaked in 72 h cultures of *P. chrysogenum pafB^paf_promoter^* compared to the *wt* strain (Figure 6A). The dramatically increased and prolonged *pafB* transcription time resulted in the detection of high amounts of secreted PAFB in the culture broth after 72 h of incubation (Figure 6B). In contrast, the use of the *paf*-terminator mildly increased the signal for *pafB*-mRNA after 48 h of cultivation but did not prolong the gene expression. No PAFB could be detected in the supernatant of this mutant (Figure 6A,B).

When comparing the biomass of the *P. chrysogenum pafB^paf_promoter^* with that of the *wt*, the dry weight was less when the strains were grown for 72–96 h in 1× PcMM than in 4× PcMM, but there was no strain-dependent difference when cultivated in either of the two media (Figure S3a). Similarly, no morphological variation from the *wt* strain could be observed (data not shown).

Interestingly, *pafB*-mRNA transcription was strongly induced in *P. chrysogenum wt* surface cultures after 72 h and 96 h, irrespective of the nutrient availability, though gene expression was slightly higher in 4× PcMM compared to 1× PcMM (Figure S3b). This indicates that PAFB might play a greater role under surface than under liquid growth conditions, where *pafB* was hardly expressed in 1× PcMM. Notably, the colonies showed intensive sporulation on 1× PcMM agar whereas sporulation of the colonies on 4× PcMM agar was delayed (Supplementary Figure S3c). This was also reflected in the conidial counts (Figure S3d). Comparison of the colonies of the *P. chrysogenum wt* and the *pafB^paf_promoter^* strain, however, showed no difference in phenotype (Figure S3a,c). This was also true, when the colony establishment of these two strains was investigated. Neither germination efficiency nor germ tube length were affected by the deregulation of the *pafB* expression (data not shown).

Taken together, our results indicate that regulatory elements in the *pafB*-promoter determine the amount and duration of *pafB* gene transcription. Deregulation of *pafB* expression does not cause any significant changes in vegetative growth, conidiation and morphology in *P. chrysogenum*.

### 3.4. The pafB-Promoter Is Strong and Inducible

Since we were able to identify conditions under which PAFB is expressed in *P. chrysogenum wt*, we tested the efficiency of the *pafB*-promoter with that of two other well established promoter-systems derived from *P. chrysogenum.* We therefore evaluated the expression of *pafB* under the regulation of the *paf*-promoter [6,20] and the xylose-inducible promoter of the xylanase gene (*xylP*) [26]. For this purpose, we used (i) the *P. chrysogenum pafB^paf_promoter^* and (ii) created a new *P. chrysogenum pafB^xylP_promoter^* mutant where the promoter of the *pafB* gene was replaced by the *xylP*-promoter. The *P. chrysogenum* mutant *pafB^xylP_promoter^* was verified to carry one single integration of this expression cassette at the *pafB* locus (Figure S1b). The performance of PAFB production in these two mutants was compared to that of the *wt* strain, in which the expression of the endogenous *pafB* was under the regulation of its own promoter. The *P. chrysogenum* mutants and *wt* strain were cultivated under the conditions that induce the respective promoters.

**Figure 7:**
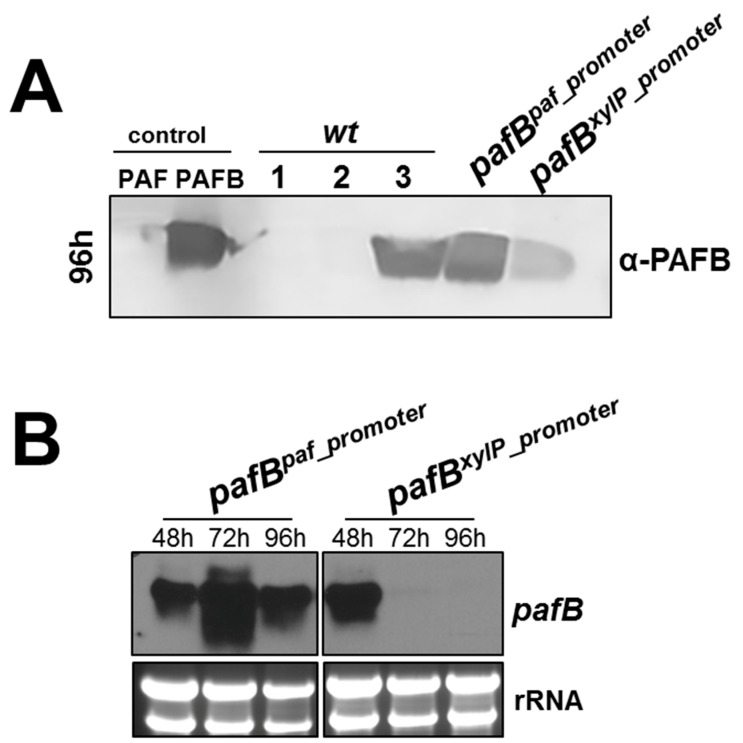
Comparison of *pafB* expression under different promoters derived from *P. chrysogenum*. PAFB expression was monitored under the *paf*-promoter, the *xylP*-promoter and the *pafB*-promoter. The mutant *pafB^paf^*^_*promoter*^ was grown in 1× PcMM (2% sucrose), the mutant *pafB^xylP_promoter^* was grown in 1× PcMM (2% xylose) and the *P. chrysogenum wt* was grown in 4× PcMM. As control the *wt* strain was grown in 1× PcMM with either sucrose or xylose as sole carbon source. (**A**) Western blot to monitor PAFB secretion into the culture supernatant after 96 h of cultivation. Culture supernatants (25 μL per lane) were loaded on a 18% (*w*/*v*) SDS polyacrylamide gel and size fractionated proteins were transferred on a nitrocellulose membrane. A polyclonal antibody was used for specific PAFB detection in a 1000-fold dilution. **1**, *P. chrysogenum wt* grown in 1× PcMM (2% sucrose); **2**, *P. chrysogenum wt* grown in 1× PcMM (2% xylose); **3**, *P. chrysogenum wt* grown in 4× PcMM (8% sucrose). As control, 1 µg purified PAFB and PAF were loaded. (**B**) Northern blot: Samples to monitor mRNA expression were taken after 48, 72 and 96 h of incubation at 25 °C. Ten μg total RNA per lane were loaded on a 1.2% denaturing agarose gel, blotted and hybridized with a *pafB*-specific DIG-labelled probe. Ethidium bromide-stained rRNA is shown as loading control.

After 96 h of cultivation, the highest PAFB-amounts in the culture broth were detected for the *P. chrysogenum wt* strain grown in 4× PcMM and the *pafB^paf_promoter^* mutant grown in 1× PcMM. The *pafB^xylP_promoter^* mutant in contrast, produced significantly less PAFB (Figure 7A).

Gene expression monitored in the time course of 48–96 h after inoculation revealed, that the *paf*-promoter efficiency was similar to that of the *pafB*-promoter in the *wt*, but superior to that of the *xylP*-promoter, as the *pafB* transcription ceased after 48 h of xylose induction (Figure 7B). These findings strengthen our assumption that the yield of PAFB in the culture supernatant depends on the amount and duration of the *pafB* transcription.

### 3.5. Secreted PAFB Exhibits Different N-Termini

For quantification of the PAFB-yield that can be achieved with the two most efficient promoters, we purified PAFB from the supernatants of 96 h old *P. chrysogenum wt* strain and *pafB^paf_promoter^* mutant. Under inducible *pafB*-promoter condition (4× PcMM) we purified up to 5 mg/L native PAFB. The *pafB^paf_promoter^* mutant grown in 1× PcMM produced up to 3 mg/L PAFB. This indicates that the protein yield from a single-integrated *pafB* gene copy regulated by the *pafB*- and the *paf*-promoter is very similar.

**Figure 8:**
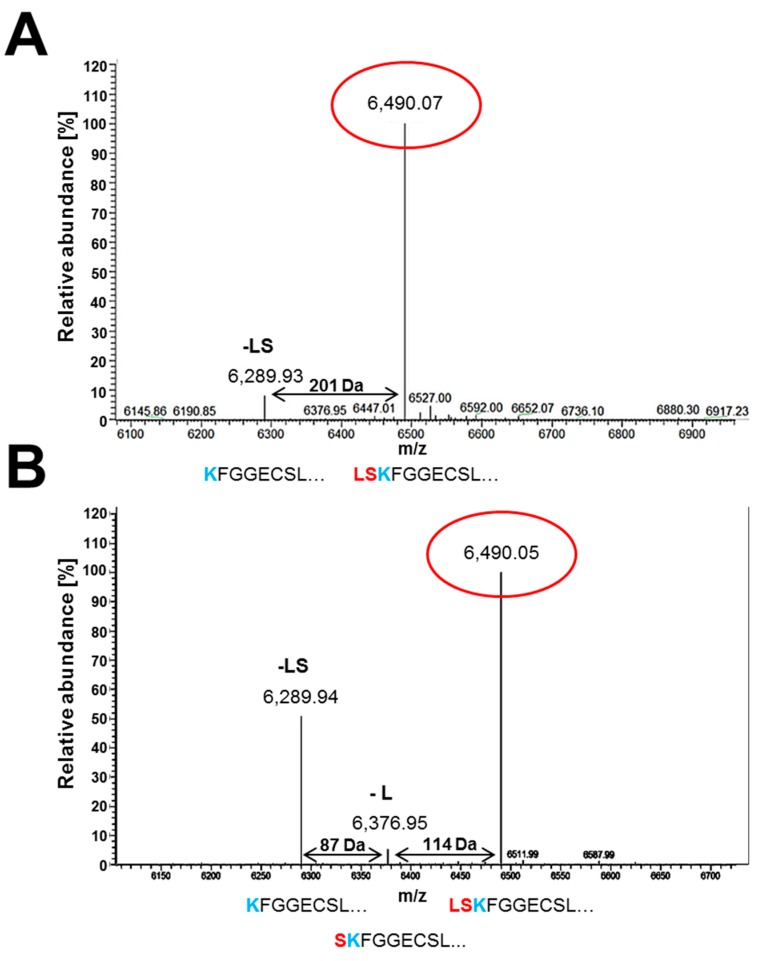
Electrospray ionization mass spectrometry (ESI-MS) analysis of purified PAFB from (**A**) *P. chrysogenum wt* and (**B**) *pafB^paf_promoter^* mutant. The *x*-axis represents the ratio between mass and charge and the *y*-axis represents the relative abundance of isotopes. The masses correspond to the different N-terminally truncated PAFB forms: the full length (6490.03 Da), the truncated form lacking the amino acid leucine (6376 Da, -L) and the truncated variant lacking two amino acids leucine and serine (6289.91 Da, -LS), respectively. The aa sequences below the peaks represent the N-terminus variations of PAFB.

In our previous study, we generated high amounts of recombinant PAFB for structural and functional studies by the multicopy integration of an expression plasmid that induces transcription of *pafB* via the *paf*-promoter [7]. Mass spectrometry identified three different N-terminal variants of PAFB, the full-length form of the mature protein and two truncated forms lacking either the last one leucine (-L) or the last two N-terminal amino acids leucine and serine (-LS) [7]. At that time, the question remained unanswered if the N-terminal variation of PAFB resulted from defective cleavage of the pro-sequence from the mature protein under overproducing conditions. We were now able to address this issue by the analysis of native PAFB produced by *P. chrysogenum wt* in 4× PcMM and the PAFB produced by the *pafB^paf_promoter^* mutant. The ESI-MS analysis indicated the presence of the full length mature protein form (6.490 kDa) and the form lacking the last two N-terminal amino acids leucine and serine (6.289 kDa), which were also found for recombinant PAFB [7]. The N-terminal variant lacking only the last N-terminal leucine (6.376 kDa) was detectable in very low amounts (Figure 8A). PAFB generated by the *pafB^paf_promoter^* mutant consisted of a mixture of all three N-terminal variants that were identified previously for recombinant PAFB (Figure 8B).

## 4. Discussion

Studies on AMPs from *A. giganteus* [21] and *P. chrysogenum* [34] suggest *afp*-like genes to be highly expressed in submerged cultures under nonfavorable growth conditions during the stationary growth phase. The *A. giganteus* AFP has been found mostly secreted under carbon starvation, heat shock and pH stress [21], PAF from *P. chrysogenum* accumulates in the culture broth under carbon limitation [34] and the gene transcription level of *anafp* is induced during environmental stress conditions [22]. Therefore, AMPs from Ascomycetes were suggested to be important molecules involved in survival under unfavorable growth conditions [25].

Our attempts to identify PAFB in the culture broth of *P. chrysogenum* when cultivated under nutrient limiting conditions had failed, so far. The existence of the gene-encoded PAFB remained questionable and we produced recombinant PAFB applying a *P. chrysogenum*-based expression system [7].

Here, we provide a comprehensive study on the culture conditions favoring the expression of the antimicrobial protein PAFB in the *P. chrysogenum wt* strain. We tested media with up to 4-fold concentrated nutrients (4× PcMM, 4× CM), where PAFB reached the highest amounts in the culture broth (Figure 2, Figure 4). To the best of our knowledge, this is the first report on the expression of a fungal AMP under nutrient excess conditions.

In comparison to PAFB, PAF was strongly induced in 1× PcMM under nutrient starvation, as reported earlier [34]. However, PAF expression was mildly repressed under nutrient excess (4× PcMM) compared to the limiting conditions, but still present in sufficient amounts. This observation points towards a more complex regulation, excluding nutrient starvation to be the only trigger for PAF expression as assumed so far [6,25] (Figure 2, Figure 4).

To further investigate the expression regulation of *pafB*, we analyzed the impact of the PcMM compounds on protein production. No protein was detectable when potassium, iron, magnesium or sucrose were added in 4-fold amounts to the 1× PcMM, respectively. Contrary, increased nitrogen availability triggered PAFB secretion. However, the protein amounts produced were significantly lower than in 4× PcMM (Figure 3). These findings suggest that the nutrient combination triggers the maximum expression of PAFB. A promoter expression analysis planned in the near future will help to dissect in detail regulatory elements involved in the *pafB* gene expression.

Interestingly, the morphology of the fungal biomass markedly differed between PAFB-inducing conditions (fluffy mycelium) and noninducing conditions (dense pellets). Some studies describe the impact of fungal morphology on the yield of fungal products [35]. It has been shown that the formation of small pellets support the production of, e.g., citric acid with *A. niger* [36], whereas enzymes like amylases or neo-fructosyl-transferases are more efficiently produced from dispersed mycelia [37]. Obviously, culture conditions that favor mycelial growth of *P. chrysogenum* enhance the PAFB production and secretion into the supernatant.

AMP expression requires distinct environmental stimuli and is under tight temporal control. Not only nutrient limitation or unfavorable growth conditions, as originally assumed, but also nutrient excess could induce the production of certain AMPs as shown in our study. It would therefore be interesting to test whether these nutrient excess conditions induce the production of other AMPs that have remained undetectable in the culture supernatant so far, e.g., AfpB from *P. digitatum* [38] or NFAP from *N. fischeri* [8].

Considering that AMPs are secreted into the supernatant of a submerse culture in the late exponential or stationary growth phase (>48 h) and importantly, in the absence of invading microbes, it is likely that AMPs have a biological function that goes beyond their antifungal activity. *P. chrysogenum* harbors more than one gene coding for AMPs [1]. The different expression regulation of PAFB and PAF let us therefore conclude that both proteins cover distinct functional roles in the producer.

For PAF an intrinsic function has been already demonstrated. The deletion of the *paf* gene resulted in a *P. chrysogenum* mutant with impaired conidiogenesis and therefore this protein was assumed to be a mediator of asexual development in *P. chrysogenum* [23]. Interestingly, apoptosis rates and expression of autophagy related genes were lower under carbon limitation in a *paf*-null mutant than in the *wt* strain [24]. Data generated via a transcriptome meta-analysis suggested a role of AnAFP during nutrient starvation and autophagic processes in *A. niger* [22].

PAFB seems to cover a function distinct from PAF, as it is produced under favorable growth conditions and during exponential growth of *P. chrysogenum*, that showed no signs of nutrient limitation or autophagy (Figure 5). Notably, our data also revealed no significant repression of ribosomal gene expression (S5 and S6), which would have been expected when autophagy was triggered by TOR signaling under PAF-inducing nonfavorable culture conditions (1× PcMM). This stands in contrast to previous reports that assign PAF a role in nutrient recycling under stress conditions [34]. Differences in cultivation conditions could explain these divergent observations, which need further investigations.

At the moment, we cannot assign a specific biological function to PAFB for *P. chrysogenum* as overproduction of PAFB resulted in no significant phenotype concerning morphology, biomass and conidiation (Figure S3). The generation of a *P. chrysogenum* strain defective in *pafB* production is in progress and will shed light on this question.

*P. chrysogenum* has been extensively used in biotechnology as “cell factory” for the production of biomolecules [39] and therefore advanced technologies for large-scale cultivation are well established. However, one of the major players in any efficient expression system is a strong promoter and the knowledge about the conditions enabling its best performance [6]. A strong promoter derived from *P. chrysogenum* is the *paf*-promoter [6]. The *P. chrysogenum*-based expression system—developed in our lab—uses the *paf*-promoter for the high yield production of AMPs in defined minimal medium for structural and functional analysis [20].

Another very prominent example for a potent fungi-derived promoter is the xylose-inducible *xylP*-promoter from *P. chrysogenum* [26,40]. This promoter has the advantage to be inducible by xylose as the sole carbon source and can be silenced by the presence of glucose. This allows the generation of biomass prior to protein expression when the promoter is silenced. Then, the mycelium can be shifted to a medium containing xylose to induce transcription and protein production. Therefore, the *xylP*-promoter is an excellent tool for the generation of products that are toxic for the producing microorganism.

In this study, we have identified a new promoter from *P. chrysogenum* with high potential for biotechnological use in protein production. The *pafB*-promoter is highly efficient when induced by nutrient excess conditions. By comparing the performance of the *pafB*-promoter to that of the *xylP*- and the *paf*-promotor in protein yield, the *pafB*-promoter turned out to be similarly efficient as the *paf*-promoter and superior to the *xylP*-promoter (Figure 7). The observed high yields of recombinant proteins achieved with the multi-copy integrations of the *paf*-promoter-based protein expression cassettes in *P. chrysogenum* [20], promises a significantly improved amount of recombinant gene products when using the *pafB*-promoter combined with multiple genomic integrations of the respective expression cassettes, but with the advantage of an inducible promoter.

Finally, the identification of culture conditions that promote PAFB expression allowed us to clarify that the variable N-termini of PAFB occur naturally and do not result from protein overexpression (Figure 8) [7]. The quantities of the respective N-terminal PAFB variants, however, differed between the respective cultivation conditions applied. It is likely that the media composition might be responsible for this observation as the amount of carbon and nitrogen, and the pH influence the presence of proteases in the culture broth [41].

## 5. Conclusions

We have identified conditions triggering the expression of the native PAFB protein in *P. chrysogenum* that differ from the reported ones and from those assumed for the so-far uncharacterized AMPs. Our results indicate that PAFB is most efficiently induced under favorable growth conditions when nutrient availability in the culture broth is high. If AMPs fulfill additional roles for their producing organisms beside their antimicrobial functions, these functions differ between AMPs originating from the same producing strain. AMP function might not be restricted to growth control by autolysis, autophagy or apoptosis under starvation conditions, as suggested so far. Further investigations are necessary to unravel these functions.

Based on the identification of culture conditions that trigger *pafB* expression, we propose that the *pafB*-promoter a perfect tool for recombinant protein expression. Furthermore, its inducibility by nutrient excess in minimal medium allows the production of toxic proteins in *P. chrysogenum*.

## Supplementary Materials

The following are available online at https://www.mdpi.com/2076-2607/7/12/654/s1. Figure S1: Scheme of construction and verification of the mutants (a) *pafB^paf_promoter^* (b) *pafB^xylP_promoter^* (c) *pafB^paf_terminator^* in comparison to the *P. chrysogenum wt*. Figure S2: Verification of penicillin production in the culture broth of *P. chrysogenum*. Figure S3: Phenotypical characterization of the *P. chrysogenum* *pafB^paf_promoter^* strain in comparison to the P. chrysogenum wt. Table S1: Media used in this study. Table S2: Microbial strains used in this study. Table S3: Oligonucleotides used in this study.
