# Supplementary material for: Nutrient Excess Triggers the Expression of the Penicillium chrysogenum Antifungal Protein PAFB
Source: Microorganisms. 2019 Dec 4;7(12):654. doi: 10.3390/microorganisms7120654 (PMC6956099; doi:10.3390/microorganisms7120654)
Supplement: Supplementary file 1 [file microorganisms-07-00654-s001.pdf]

Supplementary Materials

Nutrient Excess Triggers the Expression of the *Penicillium chrysogenum* Antifungal Protein PAFB

Anna Huber, Hannah Lerchster and Florentine Marx *

Institute of Molecular Biology, Biocenter, Medical University of Innsbruck, Innrain 80-82, 6020, Innsbruck, Austria

* Correspondence: florentine.marx@i-med.ac.at

Supplementary methods

1. Purification of Native PAFB from the *P. chrysogenum wt*

2×10^8 conidia of *P. chryosogenum wt* and the mutant *pafB*$^{paf\_promoter}$ were inoculated in 5×200 mL fourfold concentrated PcMM medium or 5×200 mL 1× PcMM medium, respectively. Cultures were grown at 25 °C for 96 h at 210 rpm. Mycelium was removed and the cell-free supernatant was ultra-filtered (Ultracell 30 kDa, Millipore, Billerica, MA, USA). The ultrafiltrate of the *wt* strain was diluted in a ratio of 1:4 with water before applied to a CM-Sepharose (Fast Flow, GE Healthcare Life Sciences, Little Chalfont, UK) column. The ultrafiltrate of the mutant *pafB*$^{paf\_promoter}$ was directly applied to the column, which was equilibrated in phosphate buffer (10 mM $NaPO_4$, 25 mM NaCl, 0.15 mM EDTA, pH 6.6).

Protein was eluted applying 0.1-0.6 M NaCl. Fractions were analyzed for the presence of PAFB and PAF by Western blot experiments. Those fractions containing only PAFB were pooled and dialyzed (3.5 K MWCO, Thermo Fisher Scientific, Waltham, MA, USA) against ultra-pure ddH_2O. Protein concentrations were determined spectrophotometrically (A_{280}) and the purity was checked by SDS-PAGE by Silver staining.

2. *P. chrysogenum* surface cultures

A conidial suspension (2×10^5/mL) was point inoculated in 5 µL aliquots on 1× PcMM or 4× PcMM agar and grown for 72-96 h at 25 °C. The colonies were removed from the agar plates using sterile tweezers and used for total RNA extraction as indicated in the main text (**Material and Methods**).

Supplementary tables

Table S1. Media Used in This Study.

Culture medium	Composition
P. chrysogenum minimal medium (PcMM)	0.3% $NaNO_3$, 0.05% $MgSO_4 \times 7H_2O$, 0.05% KCl, 0.005% $FeSO_4 \times 7H_2O$, 2% D(+)-sucrose (*w/v*), 25mM KPO_4-buffer (pH = 5.8), 0.1% trace elements A (*v/v*)
Trace elements A	0.1% $FeSO_4 \times 7H_2O$, 0.9% $ZnSO_4 \times 7H_2O$, 0.04% $CuSO_4 \times 5H_2O$, 0.01% $MnSO_4 \times H_2O$, 0.01% H_3BO_3, 0.01% $Na_2MoO_4 \times 2H_2O$ (*w/v*)
Complete medium (CM)	2.0% salt solution A, 0.1% trace elements B (*v/v*), 2.0% D(+)-glucose (*w/v*), 0.2% bacteriological peptone, 0.1% yeast extract, 0.1% NZ-Amine (*w/v*), pH = 6.5
Salt solution A	2.6 % KCl, 2.6% $MgSO_4 \times 7H_2O$, 7.6% KH_2PO_4 (*w/v*), 0.2% chloroform (*v/v*)
Trace elements B	1.3% $ZnSO_4 \times 7H_2O$, 0.07% $CuSO_4 \times 5H_2O$, 0.1% $MnSO_4 \times H_2O$, 0.006% $Na_2B_4O_7 \times 10H_2O$, 0.13% $Na_2MoO_4 \times 2H_2O$, 0.23% $FeSO_4 \times 7H_2O$ (*w/v*)
Lysogeny broth medium (LB)	1.0% NaCl, 1.0% bacteriological peptone, 0.5% yeast extract (*w/v*)

Table S2. Microbial Strains Used in This Study.

Strain	Source
Penicillium chrysogenum Q176 (wild type strain)	ATCC 10002

P. chrysogenum paf	Sonderegger et al. 2016 [1]
P. chrysogenum pafB	Huber et al. 2018 [2]
Penicillium chrysogenum pafB$^{paf\_promoter}$	This study
Penicillium chrysogenum pafB$^{paf\_terminator}$	This study
Penicillium chrysogenum pafB$^{xylP\_promoter}$	This study
Bacillus subtilis	ATCC 6633

Table S3. Oligonucleotides Used in This Study.

**Oligo name**	**Sequence 5´ 3´**
pafB3´*Not*I_rev	CAGGATGCGGCCGCTCGGTATCTTCGATAATTC
*pafB_Bgl*II_fw	AGATCTATGCATATTACTAGCATTGCCATTGTC
Onat1	CGCCGGTACGCGTGGATCGC
Onat2	AGGCACTGGATGGGTCCTTCAC
pafB5_*BamH*I_rev	GGTGGAGGATCCGTCTTGTAGAGGATTGCGG
pafB5_*Pst*I_fw	CAAGGACTGCAGCCGTTGACTAGACCTACACGC
5´pafb fl nested_fw	GCCAAGTTGCTCTTCTGATCTTCCG
3´pafb flanken nested_rev	GTGTCGGAAGTCGGGGAGC
Xylp_*Xba*I_rev_B	ATCTTCTAGACGACGGAAGCGCGCAGTCGG
Xylp_*Bgl*II_fw_B	ACATAGATCTGGTTGGTTCTTCGAGTCGATG
pafB_*Sma*I_rev	CCCGGGTCAAACTGGGGTCTGGCAG
3´paf_*BamH*I_rev	GCCGCTGGATCCCTAGTGCAGCAGTTTGATAG
pafB3_*Xba*I_fw	ACCAATTCTAGAGTTCCGCAAGAAACAGAGTCC
opafB_without prepro_fw	CTTAGTAAATTCGGAGGA
opafB_rev2	TCAAACTGGGGTCTGGCAG
opaf_without prepro_fw	CTGGCCAAATACACCGGA
opaf_rev	GATCGGATCCCTAGTCACAATCGACAGC
opafb_fw	ATGCATATTACTAGCATTGC
40S rib_protein_S6_rev	GAAGCTCAACATCTCCTACCCG
40S rib_protein_S6_fw	TACGCAGCATCACGGGCAGTC
40S rib_protein_S5_rev	CAAGGAGGTTCTGGCCGAGCA
40S rib_protein_S5_fw	TTGGCGGCGTTGATCAGCTC

Supplementary figures

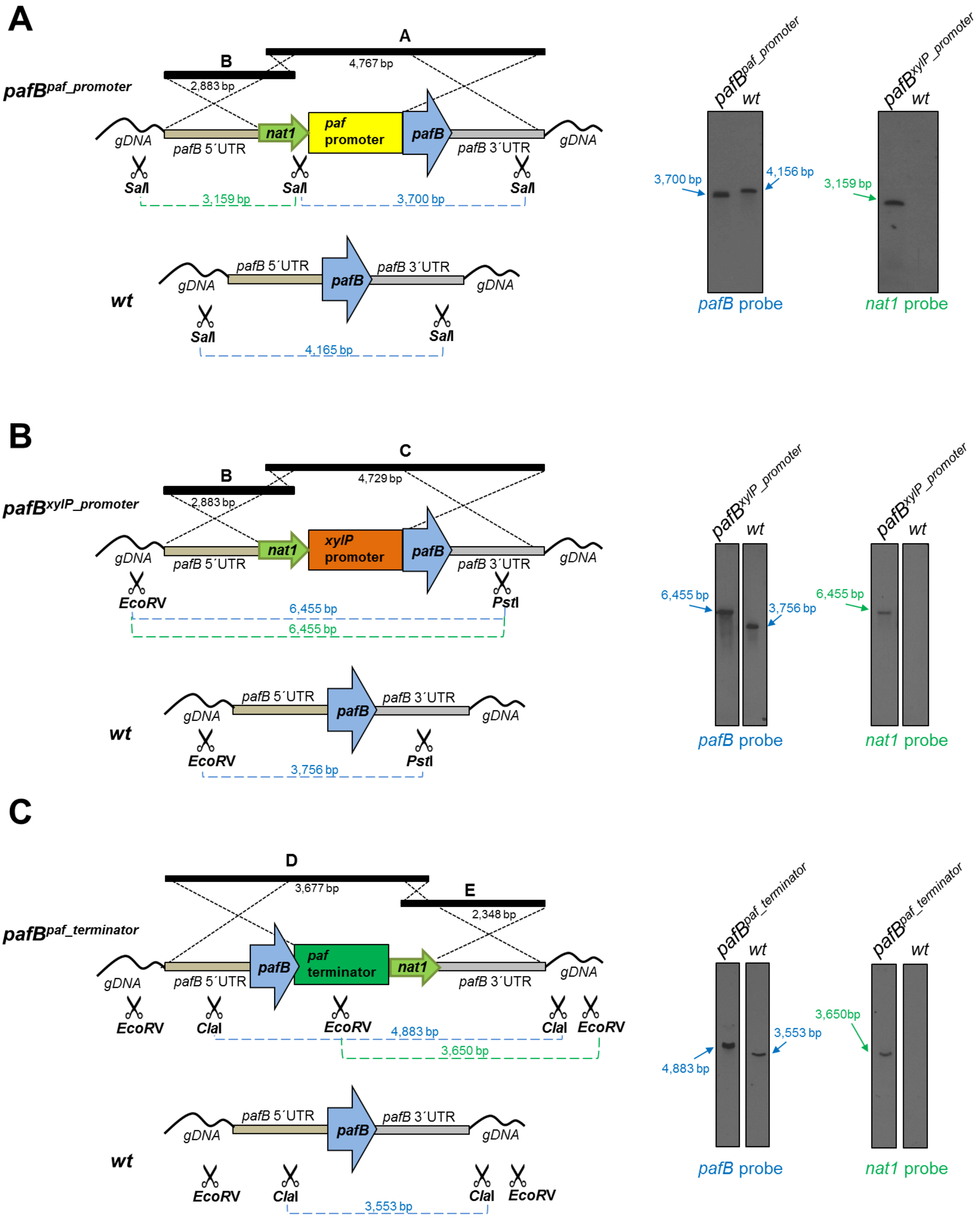

Figure S1. Scheme of construction and verification of the mutants (a) pafB^{paf_promoter} (b) pafB^{xylP_promoter} (c) pafB^{paf_terminator} in comparison to the *P. chrysogenum wt*. Left: The beige and grey boxes represent the *pafB* 5´or 3´UTR, respectively. The light green arrow and the blue arrow reflect the nourseothricin-acetyltransferase gene *(nat1)* and the *pafB* gene. The yellow, orange and dark green boxes represent the *paf*-promoter, *xylP*-promoter and *paf*-terminator, respectively. The black lines A, B, C, D and E represent the transformation fragments. The crosses show regions involved in homologous recombination. The scissors indicate the position of digestion by respective restriction enzymes used for Southern blot analysis. The dashed blue and green lines represent the expected fragments detected in Southern blot analysis by the use of a *pafB*-specific or a *nat1*-specific DIG-probe, respectively. Right: Southern blot analysis of the respective mutants compared to the *P. chrysogenum wt*. Results for *pafB*-specific and *nat1*-specific probe are indicated in blue or green, respectively.

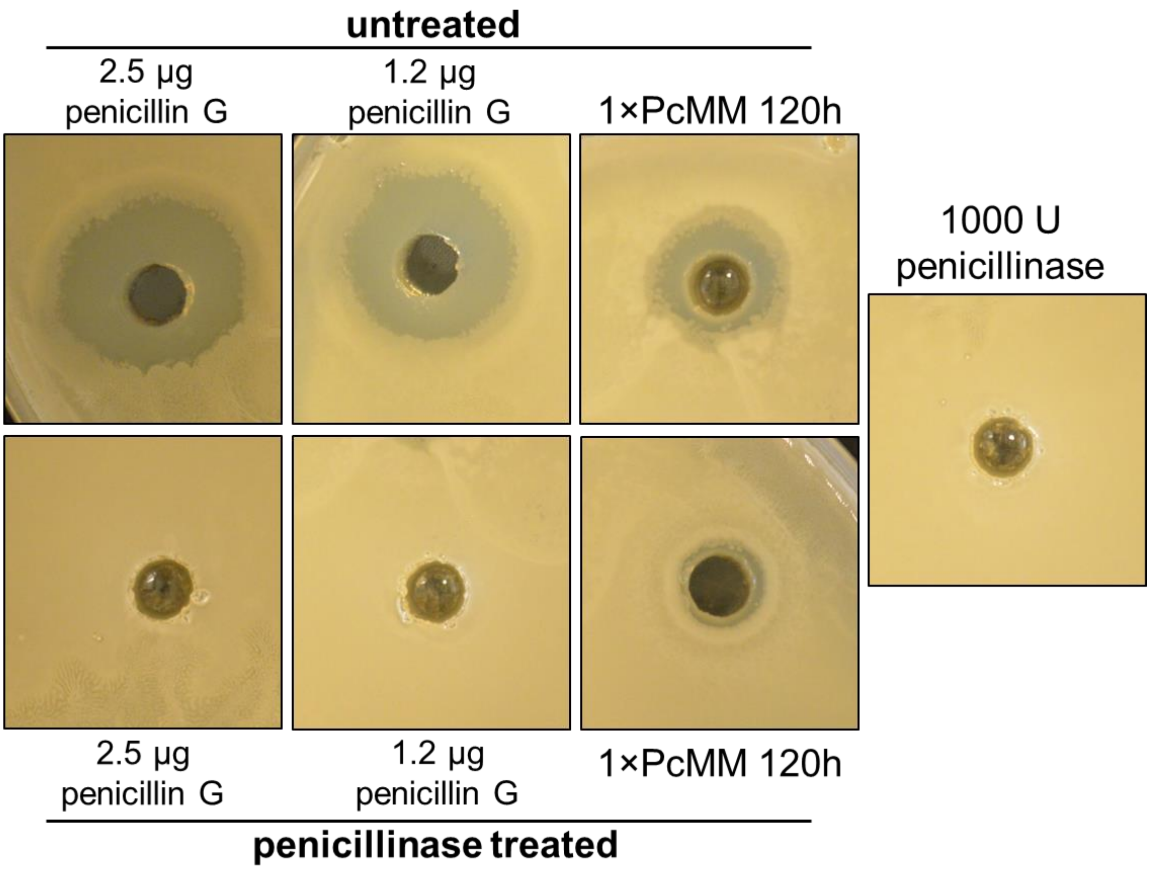

Figure S2. Verification of penicillin production in the culture broth of *P. chrysogenum*. Penicillin (2.5 µg and 1.2 µg) and 250 µL of 120 h old *P. chrysogenum* conditioned cell-free culture broth were used in an inhibition zone assay using *B. subtilis.* Samples treated for 1 h with 1000 U penicillinase. As controls, untreated samples and 1000 U penicillinase alone were included in the assay.

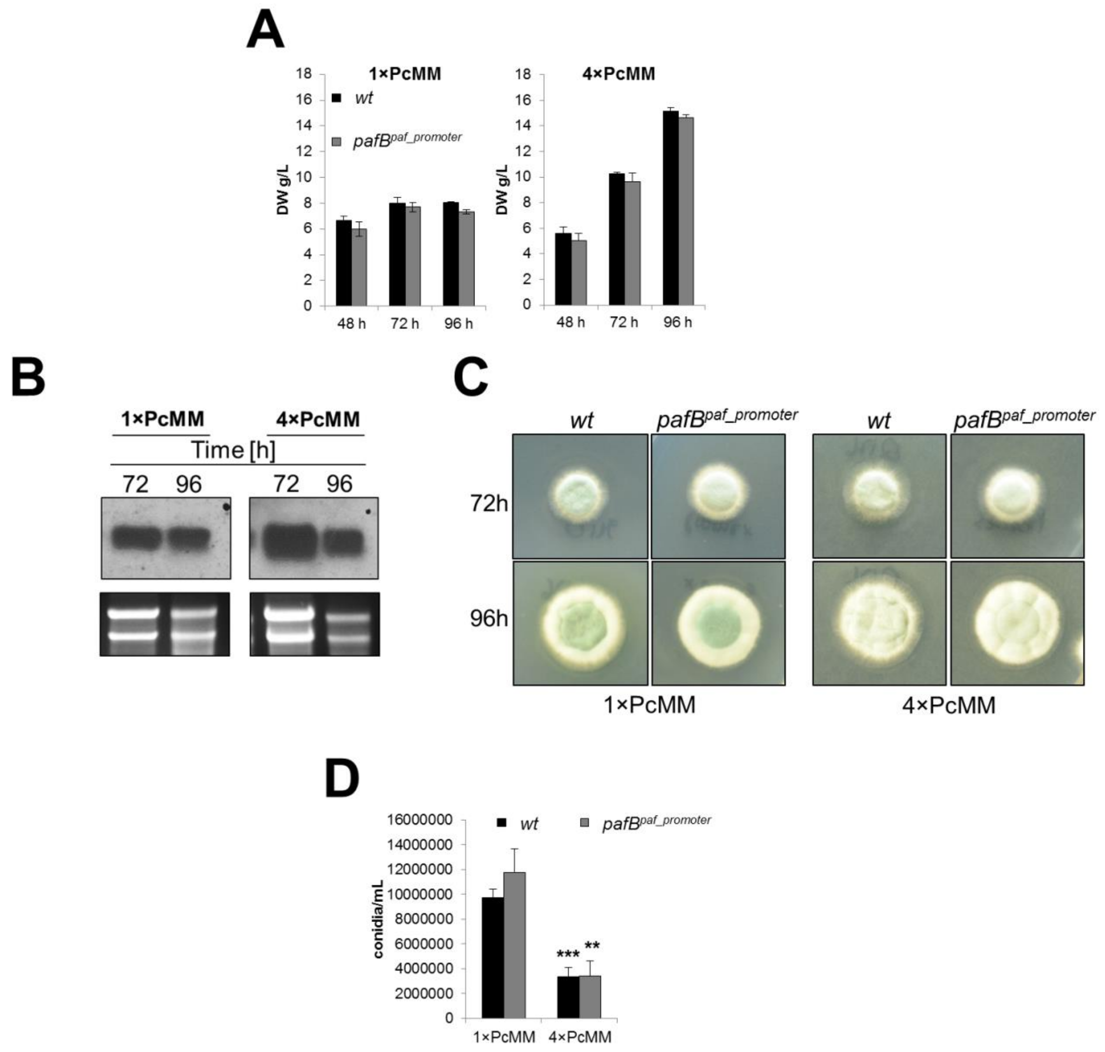

Figure S3. Phenotypical characterization of the *P. chrysogenum pafB*$^{paf\_promoter}$ strain in comparison to the *P. chrysogenum wt*. **(a)** Mycelial dry weight [DW g/L] of fungal biomass grown in liquid 1× PcMM or 4× PcMM analyzed after 48, 72 and 96 h of cultivation. **(b)** *pafB* mRNA-expression in surface cultures of *P. chrysogenum wt.* **(c)** Phenotype of *P. chrysogenum wt* or *pafB*$^{paf\_promoter}$ on 1× PcMM or 4× PcMM agar after 72 h or 96 h incubation at 25°C. **(d)** Number of conidia per mL. Strains were grown on 1× PcMM or 4× PcMM agar for 96 h and conidia were harvested and counted. Values are given as mean ± SD (n=3). P-values in **(d)** were determined to compare the number of conidia generated under 1× PcMM and 4× PcMM conditions, for the *wt* and the *pafB*$^{paf\_promoter}$ strain, respectively. ** $p \leq 0.005$, *** $p \leq 0.0005$.

References

1. Sonderegger C., Galgóczy L., Garrigues S., Fizil Á., Borics A., Manzanares P., Hegedüs N., Huber A., Marcos J.F., Batta G., Marx F. A *Penicillium chrysogenum*-based expression system for the production of small, cysteine-rich antifungal proteins for structural and functional analyses. *Microb Cell Fact* **2016**, *15*, 192. doi:10.1186/s12934-016-0586-4
2. Huber A., Hajdu D., Bratschun-Khan D., Gáspári Z., Varbanov M., Philippot S., Fizil Á., Czajlik A., Kele Z., Sonderegger C., Galgóczy L., Bodor A., Marx F., Batta G. New antimicrobial potential and structural properties of PAFB: A cationic, cysteine-

rich protein from *Penicillium chrysogenum* Q176. *Sci Rep* **2018**, *8*, 1751. doi: 10.1038/s41598-018-20002-2
